# Cost-effectiveness of nurse-delivered cognitive behavioural therapy (CBT) compared to supportive listening (SL) for adjustment to multiple sclerosis

**DOI:** 10.1186/s13561-017-0172-4

**Published:** 2017-10-10

**Authors:** I. Mosweu, R. Moss-Morris, L. Dennison, T. Chalder, P. McCrone

**Affiliations:** 1 King’s Health Economics, Institute of Psychiatry, Psychology & Neuroscience, King’s College London, Box 024, The David Goldberg Centre, De Crespigny Park, Denmark Hill, London, SE5 8AF UK; 2 Department of Psychology, Institute of Psychiatry Psychology & Neuroscience, King’s College London, London, UK; 3 School of Psychology, University of Southampton, Southampton, UK; 4 Department of Psychological Medicine, King’s College London, Cutcombe Road, London, SE5 9RJ UK

**Keywords:** Multiple sclerosis, Cost-effectiveness, Costs, Cognitive behavioural therapy, Distress, Anxiety

## Abstract

**Background:**

Cognitive Behavioural Therapy (CBT) reduces distress in multiple sclerosis, and helps manage adjustment, but cost-effectiveness evidence is lacking.

**Methods:**

An economic evaluation was conducted within a multi-centre trial. 94 patients were randomised to either eight sessions of nurse-led CBT or supportive listening (SL). Costs were calculated from the health, social and indirect care perspectives, and combined with additional quality-adjusted life years (QALY) or improvement on the GHQ-12 score, to explore cost-effectiveness at 12 months.

**Results:**

CBT had higher mean health costs (£1610, 95% CI, −£187 to 3771) and slightly better QALYs (0.0053, 95% CI, −0.059 to 0.103) compared to SL but these differences were not statistically significant. This yielded £301,509 per QALY improvement, indicating that CBT is not cost-effective according to established UK NHS thresholds. The extra cost per patient improvement on the GHQ-12 scale was £821 from the same perspective. Using a £20,000, threshold, CBT in this format has a 9% probability of being cost effective. Although subgroup analysis of patients with clinical levels of distress at baseline showed an improvement in the position of CBT compared to SL, CBT was still not cost-effective.

**Conclusion:**

Nurse delivered CBT is more effective in reducing distress among MS patients compared to SL, but is highly unlikely to be cost-effective using a preference-based measure of health (EQ-5D). Results from a disease-specific measure (GHQ-12) produced comparatively lower Incremental Cost-Effectiveness Ratios, but there is currently no acceptable willingness-to-pay threshold for this measure to guide decision-making.

## Background

The clinical effects of multiple sclerosis (MS) and subsequent ambulatory complications associated with managing the condition may have psychological effects, which may not necessarily be treated with drugs. MS patients report very high levels of emotional distress and levels of major depression as high as 20% annually and 50% over a lifetime [1–3]. Depression in MS patients has been linked to disability, negative effects on quality of life, self-harming tendencies, as well as low adherence, which may eventually result in the discontinuation of disease modifying therapies (DMTs) [4, 5]. However, elevated levels of distress or comorbid psychological disorders in MS are often overlooked in treatment [6]. There are particularly stressful moments in the trajectory of the illness (such as MS diagnosis, relapse, disease progression) and other critical life changing events when elevated levels of distress are to be expected [7].

Treating depression appears to improve adherence to Interferon beta-1B [8], and cognitive behavioural therapy (CBT) is increasingly being used to manage symptoms and enhance psychosocial outcomes for people with chronic conditions [7, 9, 10]. In MS, there is evidence that CBT may be helpful in reducing depression, anxiety, fatigue, disability, problems in dealing with cognition in addition to improving quality of life [7, 10–14]. A recent meta-analysis suggested psychological interventions (most of which were CBT-based) were at least moderately effective in treating depression in MS [14]. To date only one trial [15] has reported on the cost-effectiveness of a psychological adjustment group therapy for MS, and due to the significant amount of missing data in the quality of life data, quality-adjusted-life-years (QALYs) were not estimated, and the Beck Depression Inventory-II was used to report a cost per point reduction on the scale of £118. Two pilot studies showed that web based CBT and SKYPE delivered mindfulness groups [16] may have the potential to be cost-effective in reducing fatigue and distress in MS respectively. However, these studies had no long-term follow up and data are preliminary.

A randomised controlled trial (RCT) of a nurse-led cognitive behavioural therapy for adjusting in the early stages of multiple-sclerosis reported that CBT is more effective in reducing distress for MS patients compared to supportive listening (SL) [17] up to one year follow up. The gains for CBT were also significantly greater for those who had clinically significant levels of distress at baseline. This paper reports on a cost-effectiveness study nested within this trial. We explored both cost-effectiveness and cost utility in the whole cohort. We also conducted a subgroup analysis of patients who entered the trial with clinically significant levels of distress.

## Methods

### Design and setting

The study was a two-arm, randomised, multicentre parallel-group controlled trial. Patients were recruited from MS centres in Hampshire and South London, and randomly assigned to receive either CBT or SL. Full details of the trial methods, intervention components and findings have been reported previously [10, 18].

### Interventions

Both interventions were delivered by general nurses specifically trained to provide eight one-on-one sessions, over a 10-week period. The sessions were delivered as a combination of two face-to-face meetings and six telephone calls.

### Self-reported outcomes

Patients completed detailed measures at baseline, 15 weeks (end of treatment), 6 and 12 months post-randomisation with 12 months being the primary outcome point. The primary outcome was adjustment (defined as psychological well-being) measured using the General Health Questionnaire (GHQ-12) [19]. The GHQ-12 uses a Likert scoring method, where high scores indicate higher distress, and to allow for a meaningful cost-effectiveness analysis, the GHQ 12 score was reported as a change score, multiplied by −1 to get an inverted score in which higher figures denote better outcomes.

Quality of life was assessed using (EQ-5D-3L) [20]. EQ-5D-3L is a brief self-reported preference-based measure which considers five dimensions of health (mobility, self-care, usual activities, pain/discomfort and anxiety/depression) each consisting of three levels of functioning (e.g. the levels for the pain dimension are no pain, moderate pain and extreme pain). This measure produces a possible 243 distinct health states ranging from 11111 (full health) to 33333 (worst) [21]. Value sets estimated from general population based studies were applied to the health states to produce preference-based scores between 0 (worse health) and 1 (full health). The measure also has a visual analogue scale ranging from 0 (the worst health you can imagine) to 100 (the best health you can imagine). Quality-adjusted-life-years (QALYS) were derived from transformed EQ-5D-3L scores, using the area-under-the-curve method [22].

### Service use and costs

Contacts with the CBT and SL interventions were centrally recorded. Self-reported six-month retrospective health and social services data were collected at baseline, six- and 12-month follow-up, using an adapted version of the Client Service Receipt Inventory (CSRI) [23]. To ensure we captured relevant services the CSRI was adapted based on available literature and expert guidance. Patients provided details of their retrospective use of medication, inpatient, out-patient appointments, laboratory tests and scans, emergency department, contact with community professionals (GPs, neurologists, etc.) and informal care including access to social welfare benefits.

Service use was combined with nationally applicable unit costs [24–26] to derive total costs. Costs were measured in UK prices (£) for 2008/09, and given the one-year time horizon, there was no need to discount either costs or effects. Interventions costs were estimated by multiplying the unit cost of nurses providing the intervention by the number of sessions attended. Informal carers are not paid for their support to patients but there is still a value to this time. Informal care cost was estimated using the unit cost of a local authority home care worker as a proxy value [24]. Productivity costs were estimated through the human capital approach, which involves applying national wage rates to days off work due to illness.

### Statistical analysis

Data were analysed using Stata 11 and patients were assessed using an intention-to-treat analysis. Missing costs and QALY data were imputed using a regression-based method adjusting for baseline variables costs, EQ-5D-3L scores, GHQ-12 score, and the type of MS. Non-parametric bootstrapping methods were used to account for non-normality in the distribution of cost data [27]. Outcomes and costs were presented as mean values with standard deviations, and compared between groups at baseline and 12-months follow-up.

The economic evaluation was conducted from both a health and social care, and societal perspectives. If one intervention had lower costs and better outcomes than the comparator, then it would be considered ‘dominant’. In the event of the intervention having higher costs and better outcomes, cost-effectiveness would be assessed using incremental cost-effectiveness ratios (ICERs).

Cost-effectiveness planes (CEP) and cost-effectiveness acceptability curves (CEAC) were created to address the uncertainty around points estimates of the ICERs. For the CEP plane, non-parametric bootstrapping was used to create a joint distribution of incremental costs and outcomes, and plotting these differences on a scatter plot (one axis represents incremental costs and the other incremental outcome), to show the probability of the intervention being (i) cost-saving and more effective, (ii) cost-saving and less effective, (iii) cost-increasing and more effective and (iv) cost-increasing and less effective. The probability of the intervention being cost-effective was explored through the use of cost-effectiveness acceptability curve (CEAC), which were created by calculating a series of net benefits for a range (£0–£60,000) of plausible values defined as a decision maker’s willingness to pay for an additional unit improvement of health outcomes (e.g. QALYs) [28].

#### Sub-group analysis

The concurrent trial found that patients who had clinically significant distress at baseline showed meaningfully greater benefits from CBT over SL than those who were not distressed [7]. We conducted a sub-group analysis exploring cost-effectiveness for the distressed only, i.e. patients who scored three and above on GHQ-12 using the GHQ 0011 scoring method [29].

## Results

### Descriptive and clinical characteristics of participants

48 participants were randomised to CBT and 48 to SL. Patient characteristics at baseline were broadly similar across groups (Table 1), except that the SL group had a slightly higher mean age (43.1 vs. 40.3); were more distressed (GHQ-12), disabled (EDSS scores), with slightly lower quality of life scores (EQ-5D-3L). The proportion (23%) reporting use of antidepressants was higher for the CBT group (25 vs. 22), and more than half (60.6%) had clinically significant distress levels.

**Table 1:** Baseline characteristics of participants

	CBT (*n* = 48)	SL (*n* = 46)
	Mean(S.D.)	Mean(S.D.)
Mean age in years (SD)	40.3 (8.6)	43.1 (10.1)
Number of female participants (%)	35 (72.9)	30 (65.2)
Number of Married/cohabiting participants (%)	30 (62.5)	24 (52.2)
Number of White British participants (%)	38 (79.2)	33 (71.7)
Number of participants with A level or higher (%)	35 (72.9)	30 (65.2)
Mean EDSS score (SD)	4.9 (1.4)	5.1 (1.0)
Mean GHQ score (SD)	14.0 (5. 5)	16.4 (6.8)
Mean EQ-5D score (SD)	0.66 (0.22)	0.60 (0.26)
Number treated for depression in the past year (SD)	12 (25)	10 (22)
Number diagnosed with relapsing remitting MS (SD)	37 (77)	36 (78)

### Service use and costs

There were minimal differences in service use between the two groups. Participants accessed a wide range of health and social care services (Table 2), but the most intensively used were the GP (84% at baseline) and MS nurse (82% at baseline), followed by neurologists (66%). The proportion with GP contacts had decreased by 12-month follow-up for both groups. The percentage in contact with neurologists and MS nurses was reduced over the follow-up period, with more reductions evident in the SL group for neurologists. Most participants reported the use of at least one medication (particularly disease modifying therapies) in both groups at baseline. Physiotherapists and alternative therapists had consistently low use over the entire study period for both groups.

**Table 2:** Patient’s use of services at baseline, six and twelve months of the evaluation period

Service	Baseline				6 months				12 months			
	CBT (n = 48)		SL (n = 46)		CBT (*n* = 45)		SL (*n* = 38)		CBT (*n* = 41)		SL (*n* = 43)	
	Users Contacts		Users Contacts		Users Contacts		Users Contacts		Users Contacts		Users Contacts	
	N (%)	Mean (S.D.)	N (%)	Mean (S.D.)	N (%)	Mean(S.D.)	N (%)	Mean(S.D.)	N (%)	Mean (S.D.)	N (%)	Mean (S.D.)
General practitioner	41 (85)	2.9 (2.6)	38 (83)	2.0 (1.1)	41 (85)	2.8 (2.1)	29 (62)	2.8 (3.8)	32 (67)	2.4 (1.8)	31 (67)	1.7 (0.8)
Neurologist	33 (69)	1.6 (1.2)	29 (63)	1.1 (0.4)	22 (46)	1.2 (0.4)	16 (35)	1.1 (0.5)	22 (46)	1.2 (0.7)	15 (33)	1.0 (0)
Other doctors (including dentist)	26 (54)	4.3 (11.7)	18 (39)	1.7 (1.1)	16 (33)	4.9 (8.1)	18 (39)	2.9 (3.3)	20 (42)	2.0 (1.8)	20 43)	1.8 (1.3)
MS nurse	39 (81)	2.3 (4.1)	38 (83)	1.5 (1.0)	28 (58)	1.4 (0.6)	22 (48)	1.7 (1.2)	23 (48)	1.6 (1.2)	24 (52)	1.4 (0.8)
Pharmacist	9 (19)	1.8 (1.1)	8 (17)	1.8 (1.2)	7 (15)	1.9 (1.1)	4 (9)	2.0 (0.8)	3 (6)	1.0 (0)	9 (20)	3.3 (6.3)
Therapist	2 (67)	5.5 (3.5)	1 (2)	3.0 (.)	5 (10)	5.0 (2.2)	3 (7)	3.7 (4.6)	1 (2)	6.0 (.)	4 (9)	6.0(3.6)
Physiotherapist	12 (46)	5.4 (8.1)	14 (30)	4.2 (3.2)	5. [10]	9.2 (11.7)	8 (17)	4.0 (2.7)	8 (17)	8.1 (5.1)	10 (22)	8.6 (9.9)
Alternative therapy	9 (41)	5.3 (4.5	13 (28)	3.0 (2.2)	7 (15)	5.4 (4.3)	10 (22	4.5 (5.1)	11 (23)	6.5 (5.6)	13(28)	9.9 (8.8)
Other community-based professionals	1 (2)	20.0 (.)	0	–	9 (19)	228.6 (340.7)	10 (22)	50.8 (39.1)	11 (23)	7.4 (7.5)	7 (15)	3.7 (3.4)
Medicine	43 (90)		33 (72)		39 (81)		33 (72)		37 (77)		37 (80)	
Hospital-based services												
In-patient (length of stay)	4 (8)	1.3 (0.5)	5 (11)	1.0 (0)	2 (4)	14.0 (15.6)	3 (7)	1.0 (0)	2 (4)	12.5 (16.3)	1 (2)	14.0 (.)
Accident and emergency	4 (8)	1.5 (0.7)	3 (7)	1.0 (0)	3 (6)	1.0 (0)	2 (4)	1 (0)	2 (4)	1.5 (0.7)	3 (7)	1.0 (0)
Investigations (blood test, MRI, x-ray, CT/CAT scan, EEG)	34 (71)	2.5 (1.6)	33 (72)	2.4(1.3)	27 (56)	2.0 (1.2)	18 (39)	2.3 (1.6)	23 (48)	2.5 (2.2)	25 (54)	1.6(0.8)
Time off work (days)	7 (15)	11 (16)	7 (15)	7 (11)	9 (19)	11 (30)	7 (15)	6 (8)	9 (19)	19 (34)	7 (15)	23 (44)
Informal care (hours per/week)	48 (100)	9 (15)	45 (98)	6 (10)	42 (88)	8 (16)	38 (83)	5 (11)	40 (83)	9 (14)	42 (91)	9 (13)

Despite low admissions (10%), the CBT group had substantially more inpatient days at 12 months. More than two thirds (71%) reported investigations (e.g. blood tests, MRI, x-rays, CT/CAT and EEGs) at baseline, but at follow-up the proportions had decreased for both groups. The mean number of tests remained the same (2.5) for the CBT group, but had decreased (1.6) for the SL group. Almost all (99%) patients reported informal care at baseline, and while this had decreased at follow-up, the mean hours/week were not so different. The mean cost of CBT was £307 compared to £306 for SL over 12 weeks. At baseline, mean service costs were fairly similar between the groups (Table 3), but higher informal care costs for the CBT group (£4378 vs. £2903), contributed to much more societal costs at baseline compared to the SL group. Over the whole follow-up period, mean costs were higher in the CBT group. Drug costs contributed the highest share to health and social care costs while informal care added the greatest proportion to societal costs. Although the number receiving informal care were similar, the CBT group had greater intensity of use hence higher costs. From the health and social care (NHS) perspective CBT had higher mean costs at follow-up compared to SL (£7331 vs. £5026), but this difference, adjusted for baseline costs (£1610) was not statistically significant (bootstrapped 95% CI, −£187 to 3771). The difference in mean costs from the societal perspective (£2871), was also not statistically significant (bootstrapped 95% CI, −£2028 to £7793).

**Table 3:** Summary of service costs and outcomes at baseline, six and twelve months

	Treatment A (CBT) n = 48			Treatment B (Supportive listening) n = 46		
Cost category	baseline	6 months	12 months	baseline	6 months	12 months
	Mean (S.D)	Mean (S.D)	Mean (S.D)	Mean (S.D)	Mean (S.D)	Mean (S.D)
Intervention	0	307 (170)	0	0	306 (148)	0
Medication (drugs)	2079 (1938)	2724 (3427)	2531 (4656)	1928 (2373)	1476 (2094)	1882 (2644)
Community services (contacts with professionals)	657 (1130)	422 (430)	483 (715)	466 (402)	305 (331)	395 (482)
Hospital services	185 (559)	133 (692)	204 (956)	96 (340)	91 (393)	119 (466)
Investigations	79 (148)	35 (77)	33 (78)	78 (123)	20 (60)	15 (43)
Total hospital and social care costs	3000 (2098)	3621 (3751)	3251 (5134)	2568 (2295)	2198 (2507)	2412 (2654)
Informal care	4378 (6970)	3807 (7463)	4162 (6352)	2903 (4550)	2192 (4993)	4064 (6242)
Productivity loss	1096 (2726)	704 (3151)	388 (1742)	719 (2471)	147 (428)	385 (1987)
Total societal costs	8473 (8212)	8132 (9036)	7802 (8819)	6190 (6183)	4538 (5605)	6862 (6922)
Social benefits	1210 (1282)	1430 (1320)	1229 (1349)	1325 (1259)	1360 (1448)	1640 (1562)
EQ-5D score	0.659 (0.215)	0.661 (0.216)	0.644 (0.267)	0.595 (0.262)	0.641 (0.198)	0.622 (0.274)
GHQ-12	13.977 (5.449)		11.289 (4.63)	16.391 (6.771)		14.422 (7.316)

### Cost-effectiveness analysis and cost-utility-analyses

At baseline GHQ-12 scores were lower for the CBT group (13.98 vs. 16.40), and at 12 months both groups showed reductions in distress but the improvement in mean scores was better for the CBT group (2.69 vs. 1.97) and the difference(1.9572) statistically significant (bootstrapped 95% CI, −5.41 to −1.05) [17]. Based on the change score, CBT produced an ICER of £821/GHQ-12 score compared to SL from the health and social care perspective, indicating that for a one-point improvement on the GHQ-12 the NHS should pay £821. The ICER from the societal perspective is £1242/GHQ-12.

QALY results were also better for the intervention group at 12 months (0.6627 vs. 0.6197) but the difference (0.0053) was not statistically significant (bootstrapped 95% CI, −0.059 to 0.103). CBT was therefore more effective at improving quality of life, but expensive, yielding an ICER of £303,774 from the health and social care perspective and £541,698 from the societal perspective.

#### Subgroup analysis of those with clinical levels of distress at baseline

Results from the subgroup analysis revealed a mean cost difference of £1362 (bootstrapped 95% CI, −781 to 3612) from the NHS perspective and £3506 (bootstrapped 95% CI, −2704 to 9611) from the wider societal perspective. A statistically significant difference in the GHQ12 score (4.257 bootstrapped at 95% CI, 1.109 to 7.521) produced a corresponding ICER of £320 from the NHS perspective and £825 from the societal perspective.

For the same analysis, a mean difference in QALYs of 0.0108 (bootstrapped 95% CI, −0.051 to 0.068) indicates that the NHS would have to pay £126,111 for an additional QALY, while the society pays £324,630. Both these figures are higher than the UK cost-effectiveness threshold, but are substantially lower than the ICER in the base-case analysis.

**Fig. 1:**
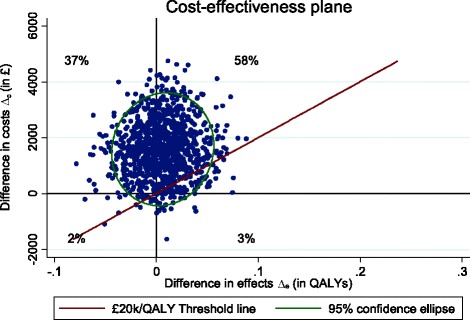
Cost-effectiveness plane of CBT compared to SL using QALYs, adjusted for baseline costs and utility, from the NHS perspective

**Fig. 2:**
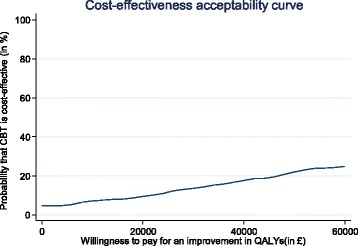
Cost-effectiveness acceptability curve of CBT compared to SL, adjusted for baseline costs and utility, from the NHS perspective

**Fig. 3:**
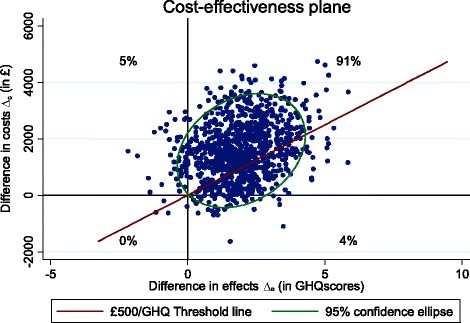
Cost-effectiveness plane using GHQ-12 change scores for CBT compared to SL, adjusted for baseline GHQ-12 score and costs, from the NHS perspective

The Cost-Effectiveness Plane (CEP) (Fig. 1) illustrates uncertainty around the estimated ICERs and it shows most (58%) of the scatter points fall on the north-east quadrant which implies that CBT produces higher costs and better QALYs. The £20,000 threshold line has very few points below it, indicating that the probability of cost-effectiveness is likely to be very low at that willingness-to-pay threshold. The CEAC (Fig. 2) demonstrates the probability of the intervention being cost-effective at varying willingness-to-pay thresholds per improvement in QALYs. Adjusted for baseline costs and utility scores, the CEAC indicates a 9% probability of CBT being cost-effective at £20,000/QALY from the health and social care perspective, which confirms the CEP findings. The second CEP (Fig. 3) produced using the GHQ-12 score and cost from a similar perspective, however show 91% of the bootstrapped ICERs falling on the north-east quadrant, indicating that CBT is better at reducing psychological distress, albeit at higher costs, compared to SL.

## Discussion

We have established that nurse-led CBT produces slightly better QALYs and is more effective in reducing psychological distress for MS, compared to SL. However, when the small effects are combined with incremental costs, CBT is not considered cost-effective compared to SL, according NICE guidelines. This conclusion was drawn from results of the ICER based on the EQ-5D-3L which show a cost per QALY of £303,774 from the health and social care perspective and £541,698 from the societal perspective. Sub-group analysis of patients meeting thresholds for clinical distress at baseline indicates a large improvement in the ICER (£126,111) from the NHS and societal (£324,629) perspectives, but still substantially higher than the recommended threshold in the UK. Results of the CEAC are also consistent with the ICER, showing only a 9% probability for CBT being cost-effective at the £20, 000 threshold when compared with SL. Comparatively, the GHQ-12 results produced much lower ICERs from both the health and social care (£821), and the societal perspective (£1242). These figures are even lower when only the distressed group is analysed; £320/GHQ-12 from the NHS perspective and £825 from the societal perspective.

The difference in the ICERs is mainly driven by incremental differences in the outcome measures, whereupon the GHQ-12 detected sizeable differences and the EQ-5D-3L produced very minimal differences. This could point to the insensitiveness of the EQ-5D-3L in patients with MS for all the five domains as illustrated in other previous work [30–32]. It is also possible that the EQ-5D-3L is unable to detect the treatment effects of psychological interventions in MS patients. This is substantiated by previous work [15], in which the EQ-5D-3L failed to detect an effect in mood for MS patients. The sample size was estimated based on the primary clinical outcome, and it is possible that it was underpowered to demonstrate a significant difference in cost-effectiveness.

These results can assist in guiding researchers and policy-makers on areas to prioritise when considering interventions for MS patients.

As far as we know, this is the first economic evaluation of CBT for adjusting to multiple sclerosis. Whilst CBT has been found to be cost-effective in other disease areas such as depression [33], our results indicate that investing in nurse-led CBT may not proffer value for money compared to the same number of sessions of nurse-led SL for MS patients.

More work needs to be done to explore the potential cost-effectiveness of CBT in MS. Providing booster sessions at follow up may improve quality of life gains as the treatment effect for CBT at the end of treatment was greater than at follow-up [17]. It is also possible that there are sub-groups within MS patients that could be targeted for future trials. It may be that future clinical trials target the therapy to those who need it most, like people with less social support or those screened and found to be distressed. Although our subgroup analysis did not produce cost-effective results, other studies [15] reported clinical and cost-effectiveness of group psychological therapy for MS patients with low mood. The control group in Humphreys et al. [15], unlike ours, received usual care (without psychological interventions), which is a closer reflection of the current reality. Most patients with MS do not receive formal therapy for their distress so it may be that compared to treatment as usual, SL or CBT could be cost effective options. More work is needed to test this hypothesis.

## Limitations

Limitations of the trial are outlined in our clinical paper [17], but the economic study had its own specific challenges. As is common in economic evaluations, there is a possibility of recall bias as participants reported on six months’ retrospective service use. There is ongoing debate relating to the appropriateness of collecting resource use data, and for this trial the most pragmatic method was self-report and the recall period was determined by the trial design. There is currently no societal value linked to a unit improvement on the GHQ-12 score, making it hard to advice on the cost-effectiveness of CBT using this measure.

We used data from a clinical trial for our economic analysis. Sample size calculations in trials are generally based on the primary clinical outcome and not costs. However, most economic evaluations (including this one) focus on probabilities rather than testing for statistical significance in cost-effectiveness. Having said that, small samples do mean that we need to be cautious in our interpretation of findings and this small sample size is a limitation. We have referred to this in the discussion.

## Conclusion

Nurse-led CBT compared to SL is not cost-effective for adjustment to MS using EQ-5D-3L but produces reasonable ICERs using GHQ-12**.** However, there is currently no acceptable willingness-to-pay threshold for this measure to guide in decision-making.

### Implications for health care provision and use, health policies, and future research

Further research in this area could be directed at designing CBT trials targeted to those who need it most, such as people with distress, as well as using alternative utility measures validated for psychological interventions in MS patients. We also know little about the cost-effectiveness of different levels of clinical expertise. In this study, senior nurses who received a two-month training course provided the CBT. It is possible that experienced clinical psychologists, although costlier, may facilitate larger treatment effects and cost savings. Finally, recent evidence [34] suggests online therapy for depression may be an effective treatment in MS. Providing minimal therapy support alongside online therapy may be a cost-effective solution.

## Abbreviations

CBT: Cognitive Behavioural Therapy
CEAC: Cost-Effectiveness Acceptability Curve
CEP: Cost-Effectiveness Plane
CSRI: Client Service Receipt Inventory
CT: Computerised Tomography
DMT: Disease Modifying Therapy
EDSS: Expanded Disability Status Scale
EEG: Electroencephalogram
EQ-5D: EuroQol Five Dimensions
GHQ-12: General Health Questionnaire
GP: General Practitioner
ICER: Incremental Cost-Effectiveness Ratio
MRI: Magnetic Resonance Imaging
MS: Multiple sclerosis
NHS: National Health Service
NICE: National Institute for Health and Care Excellence
QALY: Quality-Adjusted-Life-Year
RCT: Randomised Controlled Trial
SL: Supportive Listening
